# Digital images and the future of digital pathology

**DOI:** 10.4103/2153-3539.68332

**Published:** 2010-08-10

**Authors:** Liron Pantanowitz

**Affiliations:** Department of Pathology, Division of Pathology Informatics, University of Pittsburgh Medical Center, Pittsburgh, PA, USA

## BACKGROUND

Digital imaging today represents more of an evolution than a revolution in pathology. In a recent Scientific American review of digital pathology, the editors point out that (1) an overhaul of pathology integrating digital images is long overdue, (2) promising techniques are allowing digital images to be manipulated in novel ways, and (3) digital pathology will in due course permit more precise diagnoses.[1] In pathology, digital images can be used to make primary diagnoses, offer second opinions (consultation), for telepathology, quality assurance (e.g. re-review and proficiency testing), archiving and sharing, education and conferencing, image analysis, research and publications, marketing and business purposes, as well as tracking (e.g. audit trail of how an image was viewed). Widespread adoption of digital pathology has been hindered not only by cost and technical factors, but also largely by the mindset of technophobic pathologists.

## DIGITAL IMAGING PROCESS

**Figure 1:**
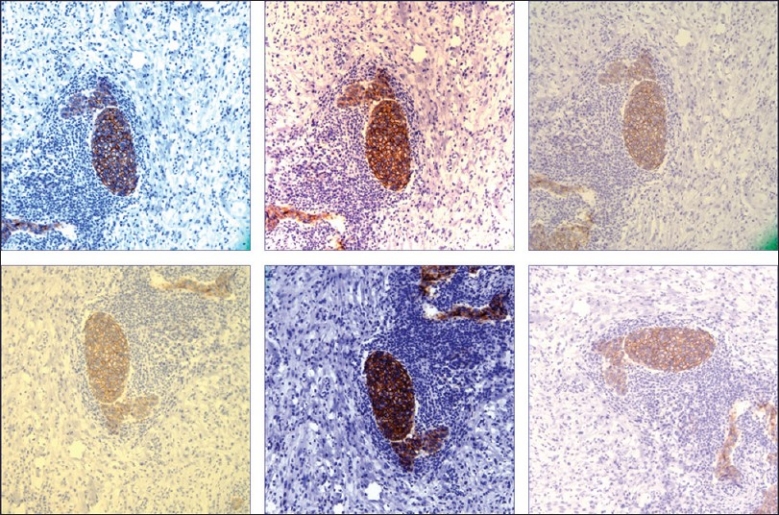
Different digital images of the same region on a glass slide photographed at the same magnification by six different pathologists, each using similar microscopes and the same attached digital cameras (HER-2/neu immunohistochemical stain)

A digital image composed of pixels represents an analog image converted to numerical form using ones and zeros (binary) so that it can be stored and used in a computer. The digital imaging process includes four key steps: (1) image acquisition (capture), (2) storage and management (saving), (3) manipulation and annotation (editing), and (4) viewing, display or transmission (sharing) of images. At present, none of these steps have been standardized. Before digital images become widely used for routine clinical work, standards are needed and the entire imaging process validated. For example, when six practicing pathologists were asked to all photograph the same region on a glass slide with similar microscopes that had the same attached digital cameras, they all provided dissimilar images [Figure 1]. Furthermore, global manipulation (e.g. contrast enhancement) of Papanicolaou test digital images has been shown to significantly affect their interpretation.[2] We also need to pay more attention to the digital pathology diagnosing station (cockpit) to ensure that they incorporate computers with sufficient performance and graphics cards, screens with excellent image resolution and color quality, as well as connectivity to the Internet, laboratory information system (LIS) and electronic medical record (EMR). The use of monitors for digital pathology should, perhaps, employ a Macbeth color checker (array of color squares) or equivalent to guarantee precise color balance.[3]

## WHOLE SLIDE IMAGING

**Figure 2:**
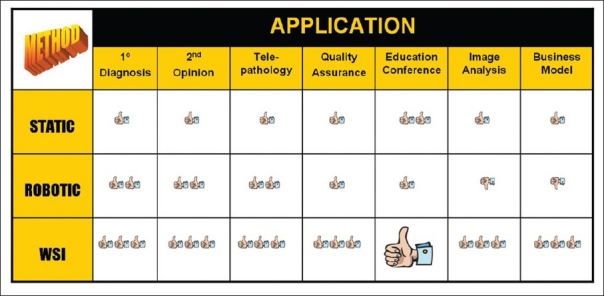
Table comparing the benefits of WSI to other modes of digital pathology. WSI gets more “thumbs up” for all applications compared to static images or live digital images viewed via robotic technology. WSI is a killer application for educational purposes

**Figure 3:**
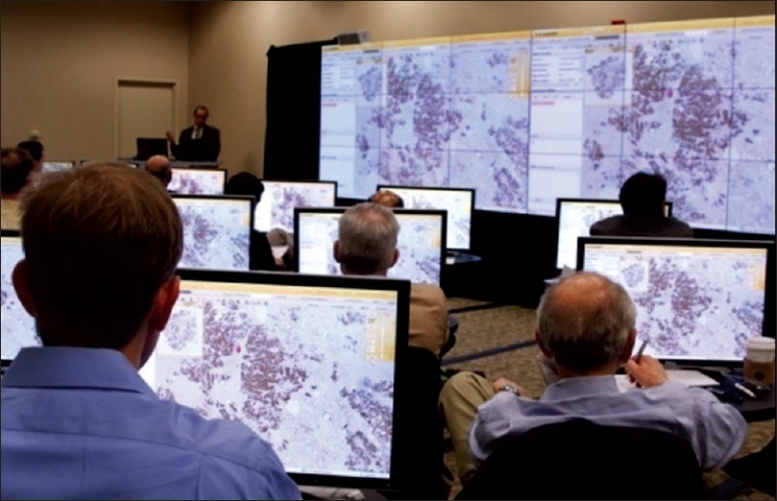
Whole slide images help create a “virtual multiheaded microscope” that supports interactive education (Image courtesy of BioImagene)

Whole slide imaging (WSI), also referred to as “virtual” or wide-field microscopy, involves digitization of glass slides, which simulates light microscopy (i.e. “digital slides”). WSI produces high-resolution digital images and involves relatively high speed digitization of glass slides of different samples (e.g. tissue sections, smears), scanning them at multiple magnifications and focal planes (*x, y* and *z* axes). Compared to static (still) and live (usually robotic) digital images, WSI is generally more beneficial [Figure 2]. For educational purposes [Figure 3], WSI are more interactive, easy to share (anywhere at anytime), involve less preparation time for conferences, provide access to the entire slide to help answer “on-the-spot” clinical questions at tumor boards, and help generate teaching sets (virtual slide boxes) that can include a wide case range and rare cases that do not fade, break or disappear. Hence, it is not surprising that WSI is increasingly being used in examinations (e.g. American Board of Pathology). WSI adoption at certain medical and dental schools has permitted them to completely abandon microscopes.[4]

**Figure 4:**
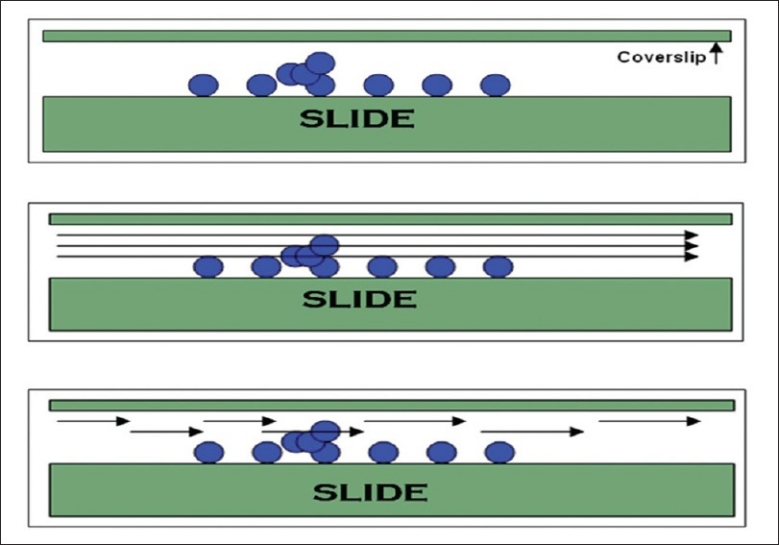
Cytology slides frequently contain 3D cell groups underneath the coverslip (top picture). The ability to view these groups in focus on a digital image can be achieved by multiplane scanning along multiple *z* axes (middle picture) or intercalation of scanned images along different focal points (bottom picture)

At present, however, even WSI is unsatisfactory to completely overcome certain limiting factors (e.g. thick smears and 3D cell groups) in cytopathology. This can be overcome by simultaneous multiplane scanning along multiple *z* axes (vertical dimension) and/or intercalation of scanned images along different focal points [Figure 4]. At present, multiplane images are technically feasible, but take a long time to scan slides and produce large files. Some investigators have overcome this problem by resorting to video microscopy (i.e. playing video images back and forward to “focus”) on cytology material.[5]

## TELEPATHOLOGY

Many interchangeable terms have been used for telepathology including digital microscopy, digital pathology, remote robotic microscopy, teleconferencing, teleconsultation, telemicroscopy, video microscopy, virtual microscopy, web conferencing, and whole slide imaging.[6] Components of a telepathology system include a digital imaging workstation to acquire images, telecommunications network to transmit images, and monitor or screen to remotely view digital images. The practice of telepathology is usually synchronous, involving two-way communication between the host and telepathologist. The history of telepathology spans approximately 40 years, highlights of which include: (1968) black and white microscopy photos were transmitted from Logan airport in Boston to the Massachusetts General Hospital; (1986) robotic telepathology was demonstrated between Texas and Washington D.C. using color video via satellite; (1989) Norway implemented a national telepathology program for frozen section services; (1994) hardware for a compete telepathology system became available; (2000) WSI comes to market; (2009) an FDA panel gathered to address approval for use of digital pathology for primary diagnosis. Today, telepathology is being employed for uses other than surgical pathology, such as telehematology and ultrastructural (digital electron microscopy) telepathology.

The three modes of telepathology currently used are: (1) *static* (store and forward) whereby pre-captured still digital images are sent via e-mail or stored on a shared server, (2) *dynamic* in which images are examined in real-time using a live telecommunications link, and (3) *hybrid* involving dynamic viewing of a static image, in which only selected areas are viewed at higher magnification. Disadvantages of static telepathology are that the telepathologist has no remote control of the glass slide(s) and has limited fields of view to examine, the host acquiring images therefore needs to have some expertise, acquiring images is labor intensive, and still images often lack clarity and/or focus. Disadvantages of robotic telepathology include a similar need for a highly experienced host (assistant), that equipment is still expensive and slow, both the host and recipient require integrated software, static image capture may not always be included with software, there is lack of interoperability between different manufacturers, high bandwidth requirements, and this set up requires significant support and ongoing maintenance. Teleconferencing (e.g. with Skype, GoToMeeting, Windows Live Messenger, Fuze, Webex) is an alternate telepathology solution that permits live, synchronous online communication between distant people.[7] Telepathology using mobile cell phones is also feasible, and has been successfully utilized for telediagnosis of malaria in remote regions of Africa.[8] There are several advantages of using WSI for telepathology such as having access to an entire digital slide, the ability to choose automated or manual scanning, high (i.e. better) resolution of images, the ability to simultaneously view images (teleconferencing), and the option to utilize added software for image management and image analysis. In a study comparing time requirements for telepathology of single block frozen sections, the turnaround time was better for WSI than robotic methods, largely because of the reduced slide interpretation time involved when viewing WSI.[9]

Many factors need to be taken into consideration when setting up telepathology. There are both direct (hardware, software) and indirect (staff, image storage) costs. Distance between the glass slide and telepathologist may be important with respect to time zones and during a downtime (i.e. will there be a pathologist close enough to be on-site in the event of a technical failure?). Technical issues may involve networks (bandwidth limitations), firewalls (that block signals or instructions for remote device control), and computers or servers that may not be enterprise compatible (e.g. due to different operating systems or antiviral software). One needs to decide how images will be managed and stored (including a retention policy), what file format(s) will be used, and if compression is acceptable. Moreover, ancillary information (e.g. patient, case, slide identification) may be in the form of barcodes or may need to be encrypted. Most importantly, practical workflow issues will need to be addressed upfront. For example, for remote frozen section diagnoses, what slides (tissue sections, smears) will be used and how will multiple/multi-specimen simultaneous frozen sections be handled? Education and the expectations of participating surgeons are equally important (e.g. what would they consider an acceptable downtime period to troubleshoot a malfunction?). Pathologist’s attitudes, perceptions, experience are also important, as is their training and ongoing evaluation of their performance for quality assurance measures.

Technical failures that have occurred during telepathology include scanning difficulties (e.g. cover slip misplacement, wet slides may stick with automatic slide feeders, variable section thickness and folds, unrecognized small pale tissue or tissue outside the cover slip, deviation between the virtual position and real position on a slide), hardware (computers, robotics) malfunction, network difficulties (e.g. freezing of video streams, relocation of systems without assigning them the correct IP address), software problems (e.g. loss of remote navigation), and image deficiencies (e.g. corrupted image, pixilated image, poor resolution, inadequate range of magnification, and poor illumination).

## IMAGE ANALYSIS

Once a digital image has been acquired, computer applications can be leveraged to analyze the information they hold. Several algorithms have been developed (e.g. pattern recognition algorithms) that promise to improve accuracy, reliability, specificity, and productivity. For example, computer assisted image analysis (CAIA) has been used to score (quantify) certain immunohistochemical stains (e.g. ER, PR and HER-2/neu breast biomarkers). In this way, CAIA gives all pathologists the same yardstick for scoring immunohistochemistry findings in breast cancer cases. This quantitative approach to tissue analysis using WSI has been referred to as “slide-based histocytometry”.[10] Multispectral image analysis is another emerging tool that exploits both spatial and spectral image information to classify images. This technology has already been shown to be valuable in certain clinical settings (e.g. cytopathology) to help differentiate and classify morphologically similar lesions.[11]

## CONCLUSION

Digital pathology is a disruptive technology, defined as a technical innovation that improves a product and/or service in a manner that the market does not anticipate. As technology becomes more cost effective, digital pathology is becoming more common. Many believe, though, that digital pathology will not take pathologists out of the “picture”.[1] At present, we have yet to see real digital slide-based routine surgical pathology in practice. With the advent of digital pathology (e.g. teleconferencing), pathologists today are beginning to interact more with each other. However, more integration of digital images with computer systems (e.g. LIS, picture archiving and communication systems or PACS) is needed, as well as standards (e.g. Digital Imaging and Communications in Medicine or DICOM) for the entire digital imaging process. Also, we need to be more mindful of emerging regulatory (e.g. CAP, FDA) and legal issues. Digital pathology has encouraged the practice of virtual pathology (separating the pathologist from the sample), allowing for new competition of services (e.g. rapid teleconsultation levels the playing field for small pathology practices). Computer-aided diagnosis of digital images is something more than the traditional microscope can offer. This technology is becoming increasingly important as anatomical pathology requires more quantitative image analysis. With these emerging imaging tools, digital pathology will undoubtedly allow pathologists to make more accurate and consistent diagnoses in the near future.

## COMPETING INTERESTS

Medical advisory board of Bioimagene.

## AUTHORS’ CONTRIBUTIONS

The author contributed solely to this paper and qualifies for authorship as defined by ICMJE http://www.icmje.org/#author
